# Do animal exhibitors support and follow recommendations to prevent transmission of variant influenza at agricultural fairs? A survey of animal exhibitor households after a variant influenza virus outbreak in Michigan

**DOI:** 10.1111/zph.12425

**Published:** 2017-11-16

**Authors:** R. J. Stewart, J. Rossow, J. T. Conover, E. E. Lobelo, S. Eckel, K. Signs, M. G. Stobierski, S. C. Trock, A. M. Fry, S. J. Olsen, M. Biggerstaff

**Affiliations:** 1 Epidemic Intelligence Service, CDC, Atlanta, GA, USA; 2 Influenza Division, National Center for Immunization and Respiratory Diseases, Centers for Disease Control and Prevention, Atlanta, GA, USA; 3 Epidemiology Elective Program, Division of Scientific Education and Professional Development, Center for Surveillance, Epidemiology, and Laboratory Services, Atlanta, GA, USA; 4 University of Georgia College of Veterinary Medicine, Athens, GA, USA; 5 Michigan State University Extension, East Lansing, MI, USA; 6 Michigan Department of Health and Human Services, Lansing, MI, USA

**Keywords:** agriculture, fairs, Influenza, prevention, swine, variant

## Abstract

Influenza A viruses circulate in swine and can spread rapidly among swine when housed in close proximity, such as at agricultural fairs. Youth who have close and prolonged contact with influenza-infected swine at agricultural fairs may be at increased risk of acquiring influenza virus infection from swine. Animal and human health officials have issued written measures to minimize influenza transmission at agricultural exhibitions; however, there is little information on the knowledge, attitudes, and practice (KAP) of these measures among animal exhibitors. After an August 2016 outbreak of influenza A(H3N2) variant (“H3N2v”) virus infections (i.e., humans infected with swine influenza viruses) in Michigan, we surveyed households of animal exhibitors at eight fairs (including one with known H3N2v infections) to assess their KAP related to variant virus infections and their support for prevention measures. Among 170 households interviewed, most (90%, 151/167) perceived their risk of acquiring influenza from swine to be low or very low. Animal exhibitor households reported high levels of behaviours that put them at increased risk of variant influenza virus infections, including eating or drinking in swine barns (43%, 66/154) and hugging, kissing or snuggling with swine at agricultural fairs (31%, 48/157). Among several recommendations, including limiting the duration of swine exhibits and restricting eating and drinking in the animal barns, the only recommendation supported by a majority of households was the presence of prominent hand-washing stations with a person to monitor hand-washing behaviour (76%, 129/170). This is a unique study of KAP among animal exhibitors and highlights that animal exhibitor households engage in behaviours that could increase their risk of variant virus infections and have low support for currently recommended measures to minimize infection transmission. Further efforts are needed to understand the lack of support for recommended measures and to encourage healthy behaviours at fairs.

## 1 | INTRODUCTION

Influenza A viruses sporadically circulate in swine (Nelson et al., 2016a) and can spread rapidly among naïve swine populations housed in close proximity, such as at agricultural fairs (Bliss, Nelson, Nolting, & Bowman, 2016; Bowman, Workman, Nolting, Nelson, & Slemons, 2014; Bowman, Sreevatsan et al., 2012; Killian et al., 2013; Nelson et al., 2016b). Agricultural fairs also provide an opportunity for bidi-rectional transmission of influenza viruses (Bowman, Sreevatsan et al.,2012) as millions of people are in close proximity to large numbers of swine (Future Farmers of America, 2016; Michigan Association of Fairs and Exhibitions, 2016), often for prolonged periods of time. When a human is infected with an influenza virus that is circulating in the swine population, it is called a variant influenza virus infection and is denoted with the letter “v.” Variant influenza viruses in humans are different from currently circulating human influenza A/H1 and A/ H3 viruses and have the potential to cause a pandemic if the virus is efficiently transmitted from person to person (Centers for Disease Control and Prevention, 2011; Reed et al., 2009).

Since novel influenza viruses, including variant viruses, became nationally notifiable in 2005, over 400 infections with variant influenza have been reported to the Centers for Disease Control and Prevention (CDC) and estimates suggest that thousands more infections may have occurred (Biggerstaff et al., 2013). Infections associated with swine exposure during fair attendance have occurred annually with the largest outbreak in 2012 (Jhung et al., 2013). Children appear to be at highest risk of variant virus infections (Epperson et al., 2013; Jhung et al., 2013) and youth who exhibit animals (“exhibitors”) at fairs may be at increased risk due to close and prolonged contact with swine over several days throughout the fair. Over 3.5 million youth engage in youth agricultural projects each year (Future Farmers of America, 2016; National 4-H Council, 2016).

To minimize variant influenza virus transmission at fairs, the National Assembly of State Animal Health Officials and the National Association of State Public Health Veterinarians (NASAHO/NASPHV) first issued *Measures to Minimize Influenza Transmission at Swine Exhibitions* in 2013. This document is updated periodically, with the most recent update in November 2016 (National Assembly of State Animal Health Officials and National Association of State Public Health Veterinarians, 2016). It includes recommendations to address activities before, during and after swine exhibitions with separate sections for exhibition organizers and youth exhibitors. Recommendations include limiting the time swine are kept on the fairgrounds to no longer than 72 hr, washing hands with soap and water whenever leaving the swine barn, restricting food and drink in animal areas, discouraging sleeping in animal areas and encouraging exhibitors to receive an annual seasonal influenza vaccine (National Assembly of State Animal Health Officials and National Association of State Public Health Veterinarians,2016). In addition, state, university and agricultural clubs in Michigan have been partnering with CDC to pilot zoonotic disease educational activities among youth participating in agricultural clubs. These are designed to improve youth exhibitor awareness and knowledge of variant influenza virus infections as well as other zoonotic diseases and encourage adoption of behaviours to help prevent zoonotic disease transmission (Centers for Disease Control and Prevention, 2017).

Although the recommendations and educational activities have been in place for several years, there is little information about the knowledge, attitudes or practice (KAP) of these measures by animal exhibitors or their families. After an August 2016 outbreak of 12 human infections with influenza A(H3N2) variant (“H3N2v”) virus associated with attendance at three fairs in Michigan (Schicker et al., 2016), we assessed the KAP and the support for these measures among house-holds of animal exhibitors.

## 2 | MATERIALS ANDMETHODS

### 2.1 | Survey design and distribution

We developed a web-based survey for households of animal exhibitors using Epi Info™ 7.2.0.1. Concurrent with the H3N2v outbreak (Schicker et al., 2016), we invited the organizers of nine fairs in Michigan to participate; organizers of one fair declined. These fairs had recently participated in enhanced monitoring for H3N2v virus infections by the state and county health departments. Fairs targeted for enhanced monitoring included those with known H3N2v virus infections and county fairs scheduled to start between 15 August 2016 and 9 September 2016 with large numbers (≥55) of swine in exhibition. Of the eight fairs that enrolled, only one had known H3N2v virus infections. Agricultural club coordinators associated with each fair sent the survey to the email addresses provided by the animal exhibitors, with instructions to have an adult in the household complete the survey. Each household received only one survey, and questions were tailored so that the survey recipient reported for all household members. The survey had four domains aimed to assess knowledge, attitudes and practices (KAP) related to variant influenza viruses as well as gauging support (indicated through checkbox) for recommendations to minimize influenza transmission from swine to people at agricultural fairs. Surveys were distributed via email and could be taken on a computer or on a mobile phone (survey in Data S1).

### 2.2 | Statistical analysis

We characterized households as associated with the fair that had H3N2v virus infections and associated with fairs that had no known H3N2v virus infections. In a secondary analysis, we also compared households with at least one swine exhibitor versus none and households where at least one member (versus none) participated in a pilot zoonotic disease educational activity targeted at youth exhibitors within the past 2 years. The unit of analysis for all characteristics and comparisons was at the household level. Following descriptive analysis, we used chi-square or Fisher’s exact tests to compare responses among respondent groups. *p* Values <.05 were considered statistically significant. All statistical analysis was conducted using SAS, version 9.3(SAS Institute Inc., Cary, NC, USA).

### 2.3 | Ethical considerations

This investigation was determined to be part of a public health response; in accordance with Federal human subjects’ protection regulations, it was not considered to be human subjects research. Participation in the survey was voluntary and anonymous.

## 3 | RESULTS

Surveys were distributed between 14 September 2016 and 5 November 2016. Among eight fairs that distributed the survey to animal exhibitor households via approximately 1,612 emails, 187 households responded for a response rate of 11.6%. Seventeen households were excluded because they reported that no household member had exhibited an animal during the 2016 fair season and thus was outside of our target population, leaving 170 respondents (Table 1). The majority of household respondents were female (86%, 146/169) and between the ages of 30 and 49 (64%, 108/169; Table 2). In total, 58 (34%) respondent households exhibited an animal at a fair with known H3N2v cases and 70 (42%) had at least one household member who exhibited swine at any fair. Reported seasonal influenza vaccine up-take, during the previous year, among all household members was 17% (28/163); among households with swine exhibitors, influenza vaccination uptake was 14% (10/69).

Animal exhibitor households reported high levels of behaviours that put them at increased risk of zoonotic diseases, including eating or drinking in swine barns (43%, 66/154) and hugging, kissing or snuggling with swine at county fairs (31%, 48/157; Table 3). These behaviours were more common among households that attended fairs with known H3N2v virus infections (Table 3) and more common among households with swine exhibitors (Table S1). Households reported washing their hands most or all times after leaving the swine barn (75%, 96/128), although 24% (41/169) reported that a hand-washing station was not readily available at their fair.

The majority of households perceived their risk of acquiring influenza from swine to be low or very low (90%, 151/167). Respondents from fairs without H3N2v infections were not significantly more likely to perceive their risk as being low or very low compared to those at fairs with known infections (93% [101/109] versus 86% [50/58], *p* = .18; Table 3) nor was there a significant difference in risk perception between households with and without swine exhibitors (87% [61/70] versus 94% [88/94], *p* = .16; Table S1).

The only recommendation that a majority of households expressly supported was the presence of prominent hand-washing stations with a person to monitor hand-washing behaviour (76%, 129/170; Table 3). Support did not significantly differ between households whose members attended fairs with and without known H3N2v infections (67% [39/58] versus 80% [90/112], *p* = .06). The measures with the least support were limiting the swine exhibit to ≤72 hr (18%, 30/170), holding swine auctions without the swine present (“distance swine auctions”; 5%, 9/170) and closing the swine barn to the public (4%, 6/170). Households whose members attended fairs with known H3N2v virus infections had significantly greater support for limiting the swine exhibition hours (29%, 17/58) and holding a distance swine auction (14%, 8/58) than households whose members attended fairs without H3N2v virus infections (12% [13/112], *p* < .01; <1% [1/112], *p* < .01). Members of households with swine exhibitors were not significantly more likely to support any of the measures than those of households without swine exhibitors (Table S1).

Households that reported at least one member participating in any pilot zoonotic disease educational activities within the past 2 years were not significantly more likely to correctly identify the definition of a zoonotic disease than those who had not participated (81% [35/43] versus 67% [76/114], *p* = .07). Results regarding prevention attitudes or practices were also not statistically different between these two groups (results not shown).

## 4 | DISCUSSION

This study provides a unique look at knowledge, attitudes, practices and support for recommended preventive measures among households of animal exhibitors at agricultural fairs. In general, members of households of animal exhibitors, including those with swine exhibitors, perceived their risk of acquiring influenza from swine to be low and regularly engaged in activities that put them at increased risk for variant influenza virus transmission, including eating and/or drinking in animal barns and coming into close contact with swine, including hugging, kissing or snuggling with a pig during the agricultural fair. With the exception of hand-washing, support for the assessed measures to minimize swine-to-human transmission of influenza was also low.

Variant virus infections occur sporadically and can result in large outbreaks. In 2012, over 300 infections were reported (Jhung et al.,2013), 16 (5.2%) hospitalizations occurred, and one death was reported. A majority (65%) of the infections in the 2012 outbreak occurred in persons with multiple days of contact with swine, which would be similar to many of the household members in this study. Our results indicate that a majority of exhibitor households participating in our survey believed they were at low risk of variant influenza virus infection. The perception of risk was higher, although not significantly, among households whose members attended fairs with known H3N2v virus infections compared to those whose members attended fairs without known infections. Risk perception was similar between households in which at least one member had participated in pilot zoonotic disease educational activities compared to households in which no members had participated. A majority of influenza A virus infections in swine present subclinically (Bowman, Nolting, Nelson, & Slemons, 2012; Bowman et al., 2017); therefore, a healthy-appearing animal may seem to present little risk to humans when in fact it may be shedding influenza virus that could cause human illness. Influenza A virus surveillance among exhibition swine during July and August 2016 detected an average 77.5% prevalence of influenza A virus infections at fairs with known H3N2v virus infections, despite few fairs reporting widespread influenza-like illness among swine (Bowman et al.,2017). This is true with other zoonotic illnesses for which exhibitors may be at risk as well. For example, in several large fair-associated out-breaks of Shiga toxin-producing *Escherichia coli* (Centers for Disease Control and Prevention, 1999, 2005, 2012a) ruminant animals such as goats and sheep were colonized with these bacteria and despite displaying no signs of illness, infected humans, causing severe illness including renal failure. Further work is needed to better educate youth exhibitors about their risk of zoonotic infections, which includes variant influenza virus infections.

While expressed support for prominent hand-washing stations was high, there was less support for the other measures to minimize the transmission of influenza viruses from swine to people, including restrictions on eating and drinking in animal barns. Less than a fifth of households supported limiting the time swine are on the fair-grounds to less than 72 hr, despite recommendations supported by a study showing the rate of influenza transmission among swine in a barn increasing dramatically after 72 hr (Bliss et al., 2016; Bowman, 2016). Initial outreach efforts to the exhibitor community to identify reasons for the lack of support for prevention measures might help human and animal health officials improve adherence to recommended measures. Once understood, health officials could build on that framework to educate exhibitors and the fair community about the risk of variant influenza virus infections and the potential benefits of the measures for reducing variant influenza virus transmission. Educational activities geared towards youth exhibitors, like those recently piloted in Michigan and elsewhere, may provide effective methods for delivering these prevention messages to those who are most affected. Effective prevention strategies should be further explored and tested to identify the best ways to increase knowledge of zoonotic diseases as well as support for activities that can limit zoonotic disease transmission.

While many studies document illness associated with attendance at agricultural fairs (Bender & Shulman, 2004; Bowman, Nelson et al., 2014; Centers for Disease Control and Prevention, 1999, 2012a,b; Schicker et al., 2016) and many groups have published prevention recommendations (Kansas Department of health and Environment; National Assembly of State Animal Health Officials and National Association of State Public Health Veterinarians, 2016; State of New Jersey, 2016), very little information exists in the literature that directly assesses behaviours and support for mitigation strategies among animal exhibitors and their households. This is a key area for continued research as interventions tailored to this population could impact a very large population at risk.

This study had limitations. This web-based animal exhibitor survey was distributed to a convenience sample of animal exhibitor households from eight fairs in Michigan that were taking part in enhanced monitoring for variant influenza virus infections during an H3N2v virus outbreak in Michigan. Emails were sent by youth agricultural club co-ordinators to their Listservs and a relatively small percentage (11.6%) completed the survey. Because of the low response rate and our limited sample of fairs, our results may not be generalizable to all animal exhibitors in Michigan or the United States. Of fairs that participated, only one had confirmed H3N2v virus infections. With the cross-sectional nature of our survey, we do not know whether differences found between fairs with and without cases occurred in response to the outbreak or were due to underlying differences in the population. Because of the need to keep the survey relatively short, we were unable to ask about all measures recommended by NASAHO/NASPHV and instead highlighted measures we considered important but not always followed. Because we were unable to identify households beyond a single email address, we could not send surveys out to individual respondents and instead asked that our survey be filled out by an adult household representative, which was often a woman aged 30–49. Therefore, what was reported from the household representative might not represent the youth exhibitor’s knowledge, activities or practices. Finally, our survey was distributed after the end of the fair season, potentially leading to recall bias in regard to exhibitor behaviours during the fair week. Non-differential bias may have occurred as those who went to a fair with known H3N2v virus infections were probably more likely to recall certain behaviours than those who went to fairs without known infections.

This study provides a unique perspective on the knowledge, attitudes and practices of animal exhibitor households surrounding variant influenza virus transmission at agricultural fairs during an ongoing outbreak of H3N2v virus infections. Household members of animal exhibitors engaged in behaviours that potentially increased their risk of acquiring variant influenza virus and in general had low support for assessed measures recommended by human and animal health officials. Health officials trying to implement prevention measures should be aware of potential barriers and work to further educate and encourage support for them among the exhibitor community. Given the lack of studies directly assessing this population, further research would be helpful to better understand attitudes, behaviours and practices of exhibitors, and to craft programmes and education targeted to this specific audience.

## Supplementary Material

Supplemental Table

Survey Tool

## Figures and Tables

**TABLE 1:** Fairs invited to participate in survey with number of survey respondents by fair

County fair	Known influenza A (H3N2)v infection	Emails sent (No.)	Survey respondents^a^ (No.)
A	No	49	0
B	No	41	9
C	No	101	19
D	Yes	430	58
E	No	200	10
F	No	72	0
G	No	60	2
H	No	659	72
Total	-	1,612	170

a Households without at least one youth animal exhibitor during the 2016 fair season excluded from results.

**TABLE 2:** Demographics of animal exhibitor household members at agricultural fairs in Michigan by attendance at a fair with or without a known influenza A (H3N2) variant (“H3N2v”) virus infection

Characteristic	Total respondents^a^	H3N2v- positive fair	Other fairs	*p*-Value
	*n* (%)^b^	*n* (%)^b^	*n* (%)^b^	
Total	170	58 (34)	112 (66)	
Age group of respondent	(*n* = 169)	(*n* = 58)	(*n* = 111)	
<18 years	9 (5)	2 (3)	7 (6)	.4
18–29 years	16 (9)	3 (5)	13 (12)	
30–39 years	36 (21)	11 (19)	25 (23)	
40–49 years	72 (43)	31 (53)	41 (37)	
>50 years	33 (20)	10 (17)	23 (21)	
Prefer not to say	2 (2)	1 (2)	2 (2)	
Sex of respondent	(*n* = 169)	(*n* = 58)	(*n* = 111)	
Male	20 (12)	7 (12)	13 (12)	1.0
Female	146 (86)	50 (86)	96 (86)	
Prefer not to say	3 (2)	1 (2)	2 (2)	
Number of household members, median (range)	(*n* = 133)	(*n* = 45)	(*n* = 88)	
	4 (1–9)	4 (2–9)	4 (1–8)	
Number of household members who attended county fair, median (range)	(*n* = 137)	(*n* = 47)	(*n* = 90)	
	4 (1–8)	4 (2–7)	4 (1–8)	
Exhibited swine at a county fair	(*n* = 166)	(*n* = 56)	(*n* = 110)	.43
	70 (42)	26 (46)	44 (40)	
All household members received seasonal influenza vaccine in preceding year	(*n* = 163)	(*n* = 57)	(*n* = 106)	<.01
	28 (17)	17 (30)	11 (10)	

a Each household surveyed provided a single survey response.

b Row percents used for “Total”; all other percents represent column percents.

**TABLE 3:** Knowledge, attitude and practices of zoonotic diseases and prevention measures among animal exhibitor household members by attendance at a fair with or without known influenza A(H3N2) variant (“H3N2v”) virus infections

Characteristic	Total	H3N2v-­positive fair	Other fairs	*p*-Value
	*n*/*N* (%)	*n*/*N* (%)	*n*/*N* (%)	
Total	170	58/170 (34)	112/170 (66)	
Correctly identifies the definition of zoonotic disease	111/157 (71)	36/53 (68)	75/104 (72)	.59
Household members report eating or drinking in swine barns	66/154 (43)	35/52 (67)	31/102 (30)	<.01
Household members report sleeping in the swine barn	2/168 (1)	1/57 (2)	1/111 (<1)	1.0
Household members report hugging, kissing or snuggling with a pig at a county fair	48/157 (31)	24/55 (44)	24/102 (24)	<.01
Household members report washing hands most or all of the time after leaving swine barn^a^	96/128 (75)	30/45 (67)	66/83 (80)	.11
Perceives risk of acquiring influenza from swine to be low or very low	151/167 (90)	50/58 (86)	101/109 (93)	.18
Supports^b^ limiting swine exhibit to ≤72 hr	30/170 (18)	17/58 (29)	13/112 (12)	<.01
Supports^b^ closing swine barn to public	6/170 (4)	2/58 (3)	4/112 (4)	1.0
Supports^b^ restrictions on eating and drinking in swine barns	85/170 (50)	25/58 (43)	60/112 (54)	.20
Supports^b^ a distance swine auction^c^	9/170 (5)	8/58 (14)	1/112 (<1)	<.01
Supports^b^ a prominent hand-washing station with monitors	129/170 (76)	39/58 (67)	90/112 (80)	.06

a Limited to those who report a known hand-­washing station at agricultural fair.

b Support measured via a checkbox on a multi-answer question—”How willing would you be to support these possible flu prevention measures for the 2017 fair season (choose all that apply).” See Q. 23 on survey in supporting material.

c Distance swine auction refers to an auction in which the swine are kept in their pens in the swine barn and exhibitors use photograph or artistic renderings of their animals to showcase them to potential bidders.

## Impacts

- Influenza viruses can spread rapidly among swine at agricultural fairs, and youth who participate in these events may be at increased risk of acquiring influenza viruses due to their close and prolonged contact with infected swine.
- Despite recommendations to minimize the transmission of influenza viruses from swine to humans, our survey found that animal exhibitors engaged in behaviours that put them at increased risk of acquiring variant influenza virus, including eating and drinking in swine barns, and hugging, kissing or snuggling with swine during agricultural fairs.
- In our survey, there was little support among households of animal exhibitors for most measures recommended to minimize transmission of influenza from swine to humans. Additional efforts to educate youth animal exhibitors about the risk of infection and benefit of prevention practices may be necessary to increase support for and adherence to recommendations.
