# Supplementary material for: Do animal exhibitors support and follow recommendations to prevent transmission of variant influenza at agricultural fairs? A survey of animal exhibitor households after a variant influenza virus outbreak in Michigan
Source: Zoonoses Public Health. Author manuscript; Available in PMC 2019 Jul 16. (PMC6631301; doi:10.1111/zph.12425)
Supplement: Supplemental Table [file NIHMS1028650-supplement-Supplemental_Table.docx]

Supplemental Table 1. Knowledge, Attitudes, and Practices of Zoonotic Diseases and Prevention Measures among Animal Exhibitor Household Members by Type of Animal Exhibited

| Characteristic  Total | Swine Exhibitor Household    n (%)  70 (42) | Non-Swine Exhibitor Household  n (%)  96 (58) | *p*-value |
| --- | --- | --- | --- |
| Correctly identifies the definition of zoonotic disease | 51/65 (78) | 59/89 (66) | 0.01 |
| Household members report eating or drinking in swine barns | 41/65 (63) | 24/86 (28) | <0.01 |
| Household members report sleeping in the swine barn | 2/69 (3) | 0/96 | 0.35 |
| Household members report hugging, kissing, or snuggling with a pig at a county fair | 42/65 (65) | 5/89 (6) | <0.01 |
| Household members report washing hands most or all of the time after leaving swine barn* | 48/61 (79) | 47/65 (72) | 0.41 |
| Perceives risk of acquiring influenza from swine to be low or very low | 61/70 (87) | 88/94 (94) | 0.16 |
| Supports** limiting swine exhibit to ≤72 hours | 15/70 (21) | 15/96 (16) | 0.34 |
| Supports** closing swine barn to public | 3/70 (4) | 3/96 (3) | 0.7 |
| Supports** restrictions on eating and drinking in swine barns | 35/70 (50) | 48/96 (50) | 1.0 |
| Supports** a distance swine auction^†^ | 5/70 (7) | 4/96 (4) | 0.5 |
| Supports** a prominent hand-washing station with monitors | 56/70 (80) | 71/96 (74) | 0.37 |

*Limited to those who report a known hand-washing station at agricultural fair

**Support measured through checking box on multi-answer question – “How willing would you be to support these possible flu prevention measures for the 2017 fair season (choose all that apply)”. See Q. 23 on survey in supporting material.

†Distance swine auction refers to an auction in which the swine are kept in their pens in the swine barn and exhibitors use photo or artistic renderings of their animals to showcase them to potentials bidders
