# Supplementary material for: Do animal exhibitors support and follow recommendations to prevent transmission of variant influenza at agricultural fairs? A survey of animal exhibitor households after a variant influenza virus outbreak in Michigan
Source: Zoonoses Public Health. Author manuscript; Available in PMC 2019 Jul 16. (PMC6631301; doi:10.1111/zph.12425)
Supplement: Survey Tool [file NIHMS1028650-supplement-Survey_Tool.docx]

Supporting Material. Animal Exhibitor Survey

**First I am going to ask you a few questions about you and your household.**

1. How old are you?

- - <18 years old
  - 18-29 years old
  - 30-39 years old
  - 40-49 years old
  - >50 years old
  - Prefer not to say

2. Are you male or female?

- - Male
  - Female

3. How many people live in your household including you? (please write number)

- - **##**

4. At which fair did you or your household members exhibit an animal? (Check all that apply)

- - Ingham County
  - Berrien County
  - Emmet Charlevoix County Fair
  - Jackson County
  - Sanilac County
  - Manistee County
  - Oceana County
  - Chippewa County
  - Allegan County
  - Other (please write-in) ­­­­­­­­­­­­­­­­­­­­­­­­­­­­­­______________________
  - No-one in our household exhibited an animal

5. How many of your household members attended the county fair? (please write #)

- - **##**

6. Has anyone in the household participated in any 4H Zoonotic Disease Prevention lessons or presentations within the last 2 years? (if no, skip to #8)

- - No
  - Yes
  - Don’t know

7. How many family members have participated? (please write number)______

8. What do you think is the definition of a zoonotic disease?

- A disease only found in zoos
- A disease that can pass between animals and humans
- A disease found in water that makes animals restless
- A disease that can pass between different animal species

**Now, I would like to ask you about interaction with pigs while at the fair**

9. Did anyone in the household exhibit pigs at a fair this year? (if yes, skip to #12)

- - No
  - Yes
  - I don’t know

10. Did anyone in the household visit the swine barn or come w/in 6 feet of a pig while visiting a fair?

- - No
  - Yes
  - I don’t know

11. Did anyone in the household touch a pig during a fair?

- - No
  - Yes
  - I don’t know

12. Did anyone in the household eat or drink in the swine barn at a county fair?

- - No
  - Yes
  - I don’t know

13. Did anyone in the household sleep in the swine barn at a county fair?

- - No
  - Yes
  - I don’t know

14. Did anyone in the household hug, kiss, or snuggle with a pig at a county fair?

- - No
  - Yes
  - I don’t know

15. Was there a hand-washing station with soap or alcohol-based sanitizer in or near the swine barn? (if no, skip to #17)

- - No
  - Yes
  - I don’t know

16. How often did members of the household wash their hands after leaving the swine barn?

- - Never
  - Some of the time
  - Most of the time
  - All of the time
  - I don’t know

**Thank you so much! You are more than halfway done! I now have just a few questions about a new text-based symptom monitoring that was used at some fairs this year.**

17. Did your household participate in the text-based symptom monitoring? (if yes, skip to #19)

- - No
  - Yes
  - I don’t know

18. If not, what is the main reason why not? (skip to #20)

- - Didn’t hear about it
  - Didn’t feel comfortable sharing information through text
  - Didn’t have a smart phone/cell phone
  - Didn’t want to pay for texts
  - Didn’t see the need
  - Don’t know

19. If yes, what was the primary reason you chose to participate?

- - Recommendation by the fair
  - Concerned about illness after hearing about sick pigs at a fair
  - Enjoy receiving reminders about health-related topics
  - I usually participate in any new and interesting tech development
  - Interested in public health
  - Other (please write in) ________________________

20. If given the opportunity, would you participate in the text-based symptom monitoring again? (if yes, skip to #22)

- - No
  - Yes
  - Unsure

21. If no or unsure, what is the main reason why not?

- I received too many text messages
- I’m not sure if it was helpful in preventing or finding illness
- When I reported an illness, I was not contacted by the health department or it took too long for the health department to contact me
- After joining, I tried to remove myself from the system and wasn’t able to
- Other (please explain)_______________________________

**Almost done! I only have few questions left about illness and prevention measures and then we will be finished. Thank you for sticking with me!**

22. In your opinion, what is the risk of you or someone in your family getting the flu from pigs at the fair?

- Very low
- Low
- Somewhat high
- Very high

23. How willing would you be to support these possible flu prevention measures for the 2017 fair season (choose all that apply)

- Limiting the time pigs are at the fair to ≤72 hours
- Closing the swine barn to everyone except swine exhibitors and their parents
- Restrictions against eating/drinking in the swine barn
- Distance swine auction (pigs stay in their barn and exhibitors present them using photos, descriptions, etc)
- Prominent hand-washing stations with monitors to remind people to wash their hands
- None of the above

24. How many of your family members (including you) got a seasonal flu vaccine (injection or nasal mist) in the past year?

- None of us got a flu vaccine last year
- Only the children <18 got a flu vaccine last year
- Some of us (adults and/or children) got a flu vaccine last year
- All of us got a flu vaccine last year

25. Did anyone in the household have a fever, cough, sore throat, runny nose, or other flu-like symptoms during the fair or in the 10 days following the fair? (if no, skip to end)

- - No
  - Yes

26. If yes, did they visit a doctor or a nurse? (if no, skip to end)

- - No
  - Yes

27. If yes, did the clinician use a swab up the nose to get a sample from the household member feeling sick?

- - No
  - Yes

28. Did a healthcare provider tell them that they had the flu?

- - No
  - Yes

Thank you for your participation in this survey!
